# Spatial structure and morphometric relationships of the deep-sea shrimp *Solenocera acuminata* (Decapoda, Solenoceridae) in the Colombian Caribbean

**DOI:** 10.3897/zookeys.1040.61005

**Published:** 2021-05-26

**Authors:** Carlos Pacheco, José Cusba, Jorge Paramo, Dante Queirolo, Daniel Pérez

**Affiliations:** 1 Facultad de Recursos Naturales Renovables, Universidad Arturo Prat, Iquique, Chile; 2 Programa de Magister en Ciencias Aplicadas mención Biología Pesquera, Universidad Arturo Prat, Iquique, Chile; 3 Pontificia Universidad Católica de Valparaíso, Escuela de Ciencias del Mar, Laboratorio de Tecnología Pesquera, Valparaíso, Chile; 4 Universidad del Magdalena, Tropical Fisheries Science and Technology Research Group (CITEPT), Santa Marta, Colombia; 5 Programa de Doctorado en Ciencias del Mar, Facultad de Ciencias Naturales e Ingeniería, Universidad de Bogotá Jorge Tadeo Lozano, Grupo de Investigación Dinámica y Manejo de Ecosistemas Marino-Costeros (DIMARCO), Santa Marta, Colombia

**Keywords:** Biomass, Caribbean, decapods, deep-sea crustacean, distribution, orange shrimp

## Abstract

Given the potential interest in targeting *Solenocera acuminata* in a new deep-sea fishery in the Colombian Caribbean, biological information is needed to support the management of this species. The objective of this study is to provide biological information about size structure, size at sexual maturity, morphometric relationships, abundances and spatial and bathymetric distribution of *S. acuminata* in the Colombian Caribbean. Specimens of *S. acuminata* were collected during four deep-sea prospecting surveys in the Colombian Caribbean Sea, which were conducted between Punta Gallinas and the Gulf of Uraba. A total of 87 exploratory fishing trawls were made between 100 and 550 m depth. Sexual dimorphism was evident, with males being smaller than females. The size at sexual maturity of the females was 95.2 mm total length (23.82 mm CL). Relatively high biomass values were found in the northern zone of the Colombian Caribbean, between Santa Marta and Riohacha. In the southern zone, higher biomass was found between Cartagena and Morrosquillo Gulf. The biomass of *S. acuminata* was higher at night (mean 1.82 kg/km^2^) than during daylight (mean 0.15 kg/km^2^). This species was distributed between 150 and 400 m depth and the highest biomass was associated with depths between 330 and 380 m. Before starting a new fishery, more research is needed to understand the life cycle parameters of deep-sea resources, such as growth, reproduction, recruitment, mortality, spawning areas and times, nursery areas and associated biodiversity.

## Introduction

Amongst decapod crustaceans, some species of the family Solenoceridae, which inhabit mostly tropical and subtropical zones, have been recognised worldwide for their importance in the development of many deep-sea fisheries (Holthuis 1980; Alves-Júnior et al. 2017; Purushothaman et al. 2017). Additionally, some shrimp of the genus *Solenocera* represent a high percentage of catches in these fisheries (Demestre and Abelló 1993; Despalatovic et al. 2006; Puentes et al. 2007; Villalobos-Rojas and Wehrtmann 2018).

*Solenocera acuminata* (Pérez-Farfante and Bullis 1973), also called “orange shrimp”, is distributed in the Caribbean Sea, including the region from the Bahamas to French Guiana, at depths between 31 and 662 m (Pérez and Kensley 1997). Although some aspects of distribution and abundance of this species have been described previously (Maynou et al. 1996; Guéguen 1997, 1998a, 2000; Charbonnier et al. 2010), detailed information about ecology and fishery-biology of this resource is lacking in other countries where the species is caught. This shrimp is endobenthic during daytime and benthic at night (Guéguen 1998a) and is only caught during the night (Charbonnier et al. 2010). This species generally inhabits the upper part of slopes (Maynou et al. 1996; Guéguen 1997, 1998a) and dense aggregations are found on muddy sediments. This species is found along the continental slope of French Guiana (western tropical Atlantic) within a very narrow bathymetric distribution (between 200 and 300 m), where *S. acuminata* is clearly dominant, reaching a maximum abundance, with average yields of 10 kg/hour by trawl (Guéguen 1997, 1998a, 2000).

Given the potential interest in *S. acuminata* for a new deep-sea fishery in the Colombian Caribbean, biological fisheries information, such as spatial distribution, growth, size structure, morphometric relationships and size at sexual maturity, is needed for an efficient fisheries management (Hilborn and Walters 1992; Haedrich and Barnes 1997; Shin et al. 2005). This allows the design and implementation of management measurements, such as protected breeding areas and fishing ban, that support sustainable use, as well as monitoring and conservation strategies (Crocos and van der Velde 1995; Ramírez-Llodra 2002; Aragón-Noriega and García-Juárez 2007). The occurrence of *S. acuminata* has been reported in the Colombian Caribbean Sea in areas, such as Magdalena, Tayrona, Palomino and La Guajira (Campos et al. 2005), with high values of occurrence frequency (41.3%), representing 2.1% in biomass and 2.1% in abundance in scientific surveys (Pérez et al. 2019). Recently, studies on the diversity of continental slope decapods and the biology of deep-sea species with potential commercial importance have been developed in the central and southern western Atlantic (Wehrtmann et al. 2012; Pérez et al. 2019). However, knowledge of some species is still quite limited. Currently, information about the biology and ecology of *S. acuminata* in the Colombian Caribbean is scarce. Most studies have reported only taxonomic records and biological information has been limited to qualitative aspects, with little information on the distribution and abundance of this species (Campos et al. 2005). The lack of knowledge on the life cycle of most deep-sea species with potential commercial interest limits the development and implementation of appropriate management measures (Villalobos-Rojas and Wehrtmann 2011). Therefore and because there are currently no management plans in place in Colombia, it is necessary to broaden our knowledge about deep-sea species and their role in the ecosystem to support their conservation and sustainable use. The objective of this study is to provide biological information about the spatial and bathymetric distribution, abundance, size structure, size at sexual maturity and morphometric relationships of *S. acuminata* in the Colombian Caribbean.

## Materials and methods

**Figure 1.**
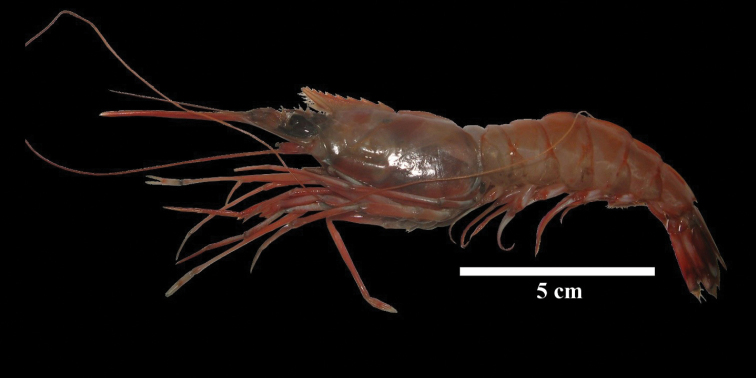
A specimen of *Solenocera acuminata* captured in the Colombian Caribbean.

**Figure 2.**
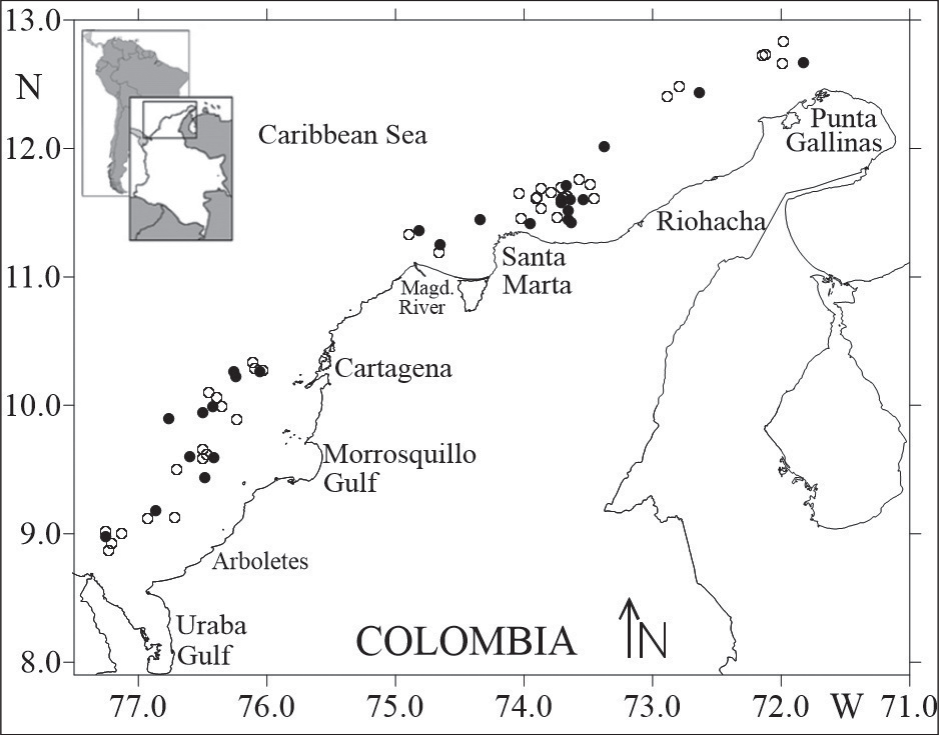
Study area in the Colombian Caribbean Sea. The white circles indicate the sampling stations and the black circles indicate the sampling station where *Solenocera acuminata* was observed.

Specimens of *S. acuminata* (Fig. 1) were collected during four deep-sea prospecting surveys in the Colombian Caribbean Sea, which were conducted between Punta Gallinas and the Gulf of Urabá (Fig. 2), in August and December 2009 and March and May 2010. Sampling was carried out onboard the commercial shrimp trawler “Tee Claude”. A Furuno FCV-1150 echo sounder with a transducer with a frequency of 28 kHz was used to identify the trawlable soft sea bottoms. A total of 87 exploratory fishing trawls were carried out in depths ranging from 100 to 550 m using a shrimp trawl with an opening of 11.6 m at the footrope and a cod-end mesh size of 44.5 mm from knot to knot. The hauls lasted 30 minutes and were conducted at an average speed of 2.5 knots, which was estimated using a Garmin Map 76CSx GPS. The swept area, which was used to calculate the catch per unit area (CPUA; kg/km^2^), was estimated from the spread of the net (11.6 m) using the vulnerability correction factor for shrimp trawl nets (0.7) (Sparre and Venema 1995) and the speed of the vessel (average of 2.5 knots) (King 2007).

**Figure 3.**
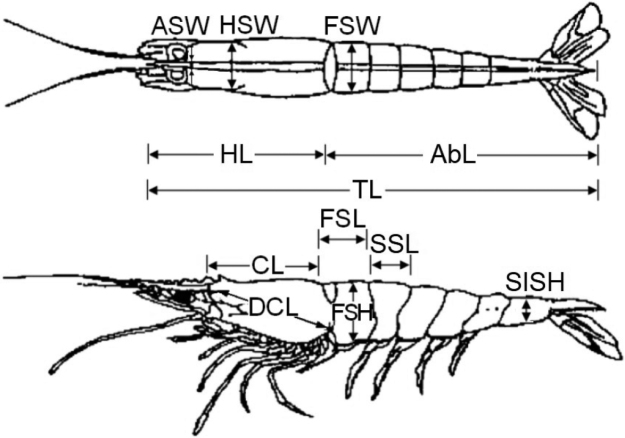
Diagram of a shrimp showing the body segments measured (Tzeng et al. 2001): total length (TL), antennal spine width (ASW), hepatic spine width (HSW), cephalothorax length (CL), diagonal cephalothorax length (DCL), first abdominal segment length (FSL), first abdominal segment width (FSW), first abdominal segment height (FSH), second abdominal segment length (SSL), sixth abdominal segment height (SISH), abdomen length (AbL) and head length (HL).

In the laboratory, the total wet weight (W) of the *S. acuminata* individuals was measured using a digital balance with an accuracy of 0.01 g. Afterwards, the samples were measured using a caliper with an accuracy of 0.01 mm, recording twelve morphometric measurements, based on previous studies developed by Tzeng et al. (2001) and Tzeng and Yeh (2002). The recorded measurements were (1) total length (TL; from the posterior margin of the ocular margin indent to the telson), (2) antennal spine width (ASW), (3) hepatic spine width (HSW), (4) cephalothorax length (CL; from the posterior margin of the ocular indent to the posterior margin of the carapace, excluding the rostrum), (5) diagonal cephalothorax length (DCL), (6) first abdominal segment length (FSL), (7) first abdominal segment width (FSW), (8) first abdominal segment height (FSH), (9) second abdominal segment length (SSL), (10) sixth abdominal segment height (SISH), (11) abdomen length (AbL; this measurement, which is used since the head is removed in fishing activities, extends from the end of the thorax to the telson) and (12) head length (HL; from the rostrum to the posterior margin of the carapace) (Fig. 3). The individuals were sexed, with males being identified by the presence of a petasma and females by the presence of a thelycum. The macroscopic maturity stage was determined for females using, as a reference, the five-stage scale (immature, early maturing, late maturing, mature and spent-recover) proposed for *Solenocera choprai* (Dineshbabu and Manissery 2008).

### Data analysis

Size structure was analysed by means of a frequency distribution, grouping the data in 5 mm intervals, for both females and males. Differences in the size frequency distribution between females and males were tested using a non-parametric Kruskal-Wallis Test (Gotelli and Ellison 2004).

The length-weight relationship was determined using the potential equation (W = *a*TL*^b^*), the parameters of which were obtained from least squares fitting, having previously performed a linearisation of the function by logarithmic transformation: ln W = ln a + b ln TL, where *W* is the total weight in g, *TL* is the total length in mm, *a* is the intercept (condition factor or initial growth coefficient) and *b* is the growth coefficient (Ferreira et al. 2008). As a measure of goodness of fit, the coefficient of determination (r^2^) was used. A 95% confidence interval was also estimated for the parameters and a Student’s t-test was conducted to determine if the sample presented isometric growth (H0: β = 3, α = 0.05). On the other hand, morphometric relationships were identified by a linear model (Y = a + bX) using least squares estimation, where (Y = TL), X = each independent variable (ASW, HSW, CL, DCL, FSL, FSW, FSH, SSL, SISH, AbL and HL) and *a* and *b* are the parameters of the equation. To evaluate the existence of possible differences between the slopes of the sexes, an analysis of covariance was performed (ANCOVA) (Zar 2009).

The analysis of the size at sexual maturity was performed by the logistic function:

$P=\frac{1}{\left(1+e^{\left(a+b*X\right)}\right)}$

where *P* is the proportion of mature females, *a* and *b* are the parameters and *X* corresponds to total length (TL) or cephalothorax length (CL). The size at sexual maturity is obtained by TL_50%_ = (-*a*/*b*) and CL_50%_ = (-*a*/*b*) (King 2007), fitting the logistics curve using a generalised linear model (GLM) with a binomial distribution and logistics link (Dobson 2002; Wheeler et al. 2009) using the GLM function in R software. The estimated TL_50%_ and CL_50%_ confidence intervals were calculated using a bootstrap procedure that randomly re-sampled the data 10,000 times, resulting in corresponding numbers for the generalised and estimated linear models of TL_50%_ and CL_50%_. The 2.5 and 97.5 percentiles of the TL_50%_ and CL_50%_ estimates were used as the confidence intervals (CI) (Haddon 2001).

The total and cephalothorax lengths were determined to be the primary measurements for the break point analysis in females and males, since these measurements are the most frequent recorded values in crustaceans (Özcan and Katağan 2011). The TL and CL were linked to the different morphometric measurements recorded for each individual (Queirós et al. 2013) to be compared with the size at sexual maturity calculated with the logistic function. A segmented regression model from the segment package in R software was used for estimating the break point; this model is based on the relationships between two explanatory variables that are represented by two straight lines connected by a break point (Muggeo 2003, 2008). The fitting was made by minimising the gap parameter, which measures the space between the two regression lines on each side of the break point. When the algorithm converges, the gap parameter approaches zero, minimising the standard error of the break point. The break point was considered an indicator of the size at the beginning of maturity for females and males, as long as the *t* value associated with the gap parameter was less than two (Muggeo 2008). In addition, the Davies Test was used to test for significant differences in slopes between fitted segments (Davies 1987; Muggeo 2008; Queirós et al. 2013; Williner et al. 2014).

The spatial distribution of the biomass (kg/km^2^) and size (CL, mm) of *S. acuminata* was determined by a geostatistical method (Rivoirard et al. 2000; Paramo and Roa 2003), in which the populations are seen as spatially stochastic processes, with the variable of interest varying randomly at any given location (Paramo and Roa 2003). First, spatial distribution was modelled using an average model for the entire sampling region of interest, based on the computed structural tool. This structural tool and model are the experimental and model variograms, respectively (Paramo and Roa 2003). Several variogram models (e.g. spherical, exponential and Gaussian models) were fitted to the experimental variogram according to the weighted least square minimisation criterion (Cressie 1993). Then, ordinary point kriging was used to interpolate the data for the not-sampled stations inside the spatial distribution area (Isaaks and Srivastava 1989). Finally, an intrinsic geostatistical method was applied to estimate the variance in the mean biomass (Rivoirard et al. 2000; see Paramo and Roa 2003 for more explanation). The spatial analysis was performed by R software (geoR library) (Ribeiro and Diggle 2001). A non-parametric Kruskal-Wallis Test was used to test for possible differences in biomass (kg/km^2^) during the day and night (Gotelli and Ellison 2004). A cumulative frequency distribution (CFD) (Perry and Smith 1994) was applied to evaluate the relationship between *S. acuminata* biomass and depth. The maximum absolute vertical distance between the curves was calculated to determine the statistical significance (P) of the difference between curves. The hypothesis of a random relationship between both CFDs was evaluated with 2000 randomisations by Monte Carlo re-sampling for the CPUA and depth (Perry and Smith 1994; see Paramo et al. 2003).

## Results

**Figure 4.**
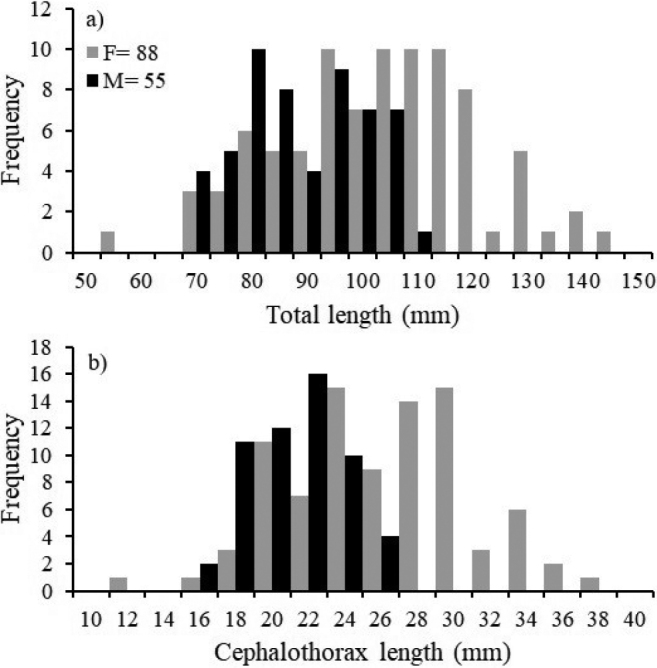
Frequency distributions of **a** total length (TL) and **b** cephalothorax length (CL) for females (F) and males (M) of *Solenocera acuminata*.

A total of 147 individuals were captured in 26 stations (Fig. 2), of which 59.9% (88 individuals) were female, 37.4% (55 individuals) were male and 2.7% (4 individuals) were indeterminate. The sizes of the females and males ranged from 56.18 to 146.70 mm TL (mean 105.95 ± 18.10 mm) and from 71.18 and 113.22 mm TL (mean 91.46 ± 11.30 mm), respectively. The females’ CL varied from 12.92 to 38.17 mm (mean 27.03 ± 4.98 mm) and that of the males between 17.33 and 27.97 mm (mean 22.17 ± 2.6 mm). The weights of the females and males fluctuated between 3.50 and 38.40 g (14.91 ± 7.63 g) and 4.30 and 15.30 g (8.44 ± 3.01 g), respectively. Statistically significant differences in sizes and weight were found between the sexes (*P* < 0.05) and sexual dimorphism was evident, with males being smaller than females (Fig. 4).

**Figure 5.**
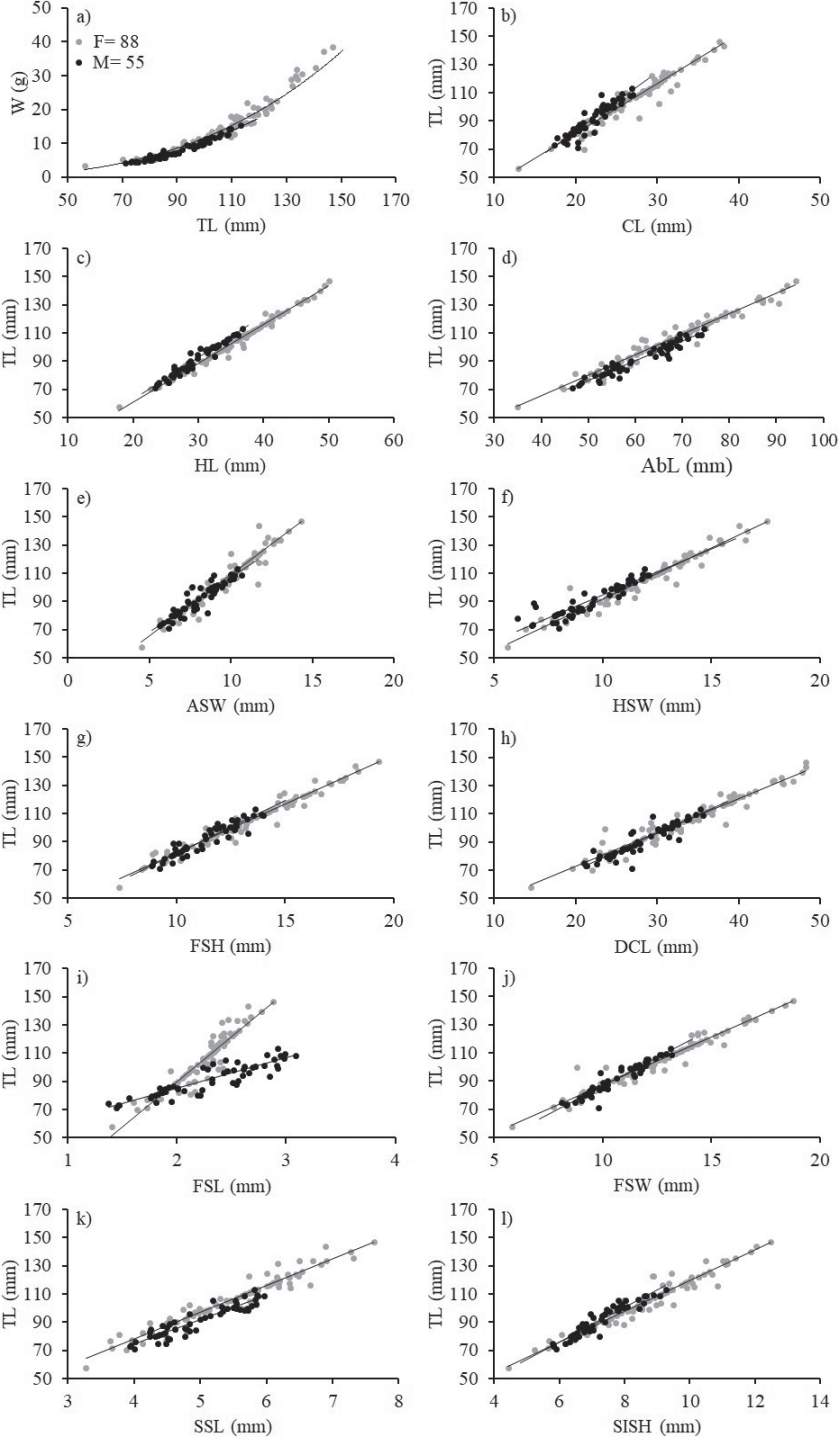
Morphometric relationships of the females (grey circles) and the males (black circles) of *Solenocera acuminata* in the Colombian Caribbean: **a** total weight (W) vs. total length (TL) **b**TL vs. cephalothorax length (CL) **c**TL vs. head length (HL) **d**TL vs. abdomen length (AbL) **e**TL vs. antennal spine width (ASW) **f**TL vs. hepatic spine width (HSW) **g**TL vs. first abdominal segment height (FSH) **h**TL vs. diagonal cephalothorax length (DCL) **i**TL vs. first abdominal segment length (FSL) **j**TL vs. first abdominal segment width (FSW) **k**TL vs. second abdominal segment length (SSL) and **l**TL vs. sixth abdominal segment height (SISH).

The analysis of the length-weight relationship indicated allometric growth (*b* ≠ 3) in females, while males showed isometric growth (*b* = 3) (Table 1). The results of the ANCOVA revealed significant differences between the slopes of females and males in the weight-length relationship (Fig. 5a, Table 1). The morphometric relationships between TL vs. ASW, HSW, CL, DCL, FSL, FSW, FSH, SSL, SISH, AbL and HL showed high coefficients of determination (r^2^ > 0.81) indicating a high correlation between sizes. The ANCOVA showed statistically significant differences between parameter *b* of females and males in linear relationships (TL vs. CL, HL, FSL and FSW). In contrast, there was no significant difference in parameter *b* (TL vs. AbL, ASW, HSW, FSH, DCL, SSL and SISH) (Fig. 5b–l, Table 2).

**Table 1.** Parameters of the relation between size and weight in female (F) and male (M) *Solenocera acuminata* from the Colombian Caribbean; *a*: intercept, *b*: the allometry coefficient, CI: confidence intervals.

Sex	N	a	a (CI 95%)	b	b (CI 95%)	r2	t - test	F	*P*-value
							(b)	(ANCOVA)	(ANCOVA)
F	88	0.00002	0.00001 to 0.00004	2.868	2.734 to 3.002	0.955	0.054	30.17	< 0.05*
M	55	0.00002	0.00001 to 0.00004	2.848	2.681 to 3.016	0.956	0.075		

* Significant (*P* < 0.05).

**Table 2.** Parameters and confidence intervals (95%) of morphometric relationships in females and males of *Solenocera acuminata*: Total length (TL), cephalothorax length (CL), head length (HL), abdomen length (AbL), antennal spine width (ASW), hepatic spine width (HSW), first abdominal segment height (FSH), diagonal cephalothorax length (DCL), first abdominal segment length (FSL), first abdominal segment width (FSW), second abdominal segment length (SSL) and sixth abdominal segment height (SISH). Degrees of freedom for all relationships = 139.

Morphometric relationship	Sex	N	a	a (C.I. 95%)	b	b (C.I. 95%)	r^2^	F (ANCOVA)	*P*-value (ANCOVA)
TL = a+b*CL	F	88	10.613	5.503 to 15.723	3.526	3.341 to 3.713	0.943	5.066	0.026*
	M	55	1.353	-8.029 to 10.735	4.064	3.644 to 4.484	0.876		
TL = a+b*HL	F	88	5.711	3.018 to 8.403	2.755	2.682 to 2.828	0.985	5.245	0.024*
	M	55	3.231	-1.961 to 8.423	2.971	2.797 to 3.144	0.957		
TL = a+b*AbL	F	88	7.488	3.496 to 11.479	1.452	1.394 to 1.509	0.967	0.441	0.508
	M	55	5.352	-0.914 to 11.618	1.416	1.313 to 1.518	0.936		
TL = a+b*ASW	F	88	22.119	16.852 to 27.385	8.674	8.141 to 9.208	0.924	3.35	0.069
	M	55	29.376	22.262 to 36.489	7.774	6.896 to 8.653	0.856		
TL = a+b*HSW	F	88	18.917	14.598 to 23.237	7.26	6.907 to 7.613	0.951	3.888	0.051
	M	55	29.622	21.993 to 37.252	6.501	5.71 to 7.293	0.837		
TL = a+b*FSH	F	88	12.828	8.784 to 16.871	6.921	6.626 to 7.217	0.962	2.095	0.15
	M	55	6.758	-1.529 to 15.046	7.517	6.787 to 8.247	0.889		
TL = a+b*DCL	F	88	24.507	20.143 to 28.871	2.413	2.286 to 2.539	0.94	1.287	0.259
	M	55	17.759	8.854 to 26.663	2.625	2.302 to 2.928	0.84		
TL = a+b*FSL	F	88	-39.069	-50.748 to -27.391	64.398	59.248 to 9.547	0.88	219.94	< 0.05*
	M	55	41.748	35.046 to 48.449	21.641	18.782 to 24.5	0.81		
TL = a+b*FSW	F	88	19.288	15.077 to 23.499	6.783	6.459 to 7.106	0.953	8.958	0.003*
	M	55	6.453	-0.612 to 13.517	8.029	7.368 to 8.692	0.918		
TL = a+b*SSL	F	88	2.839	-3.214 to 8.892	18.833	17.743 to 19.923	0.932	2.012	0.158
	M	55	5.067	-4.155 to 14.289	17.353	15.514 to 19.192	0.871		
TL = a+b*SISH	F	88	10.115	4.845 to 15.385	10.923	10.333 to 11.514	0.94	3.856	0.052
	M	55	1.694	-6.316 to 9.704	12.362	11.266 to 13.457	0.906		

* Significant (*P* < 0.05).

**Figure 6.**
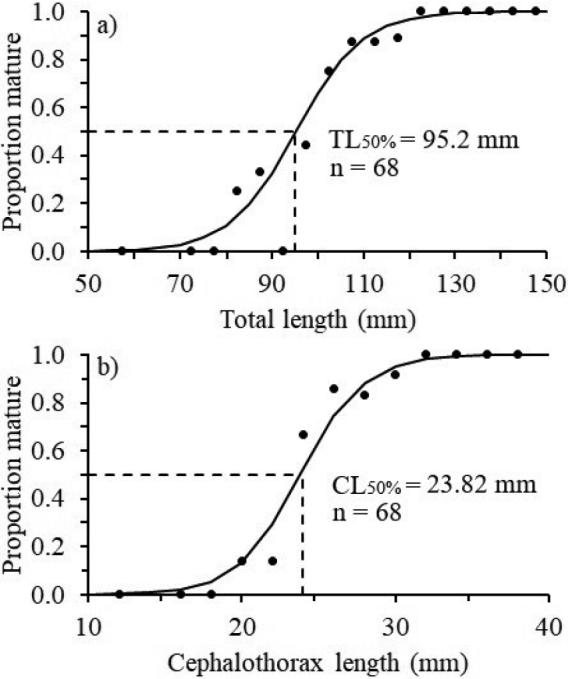
**a** Size at sexual maturity and total length (TL_50%_) and **b** cephalothorax length (CL_50%_) of *Solenocera acuminata* females in the Colombian Caribbean.

The size at sexual maturity was calculated with a total of 68 females (34% immature and 66% mature). The size at sexual maturity (TL_50%_) of females was 95.2 mm TL (95% CI = 94.22–96.77) and 23.82 mm CL (95% CI = 23.6–24.2) (Fig. 6). The parameters of the logistic model of TL_50%_ were as follows: *a* = 13.25 and *b* = 0.14; for the logistic model of CL_50%_, *a* = 11.59 and *b* = 0.49; for both cases, r^2^ = 0.99.

**Figure 7.**
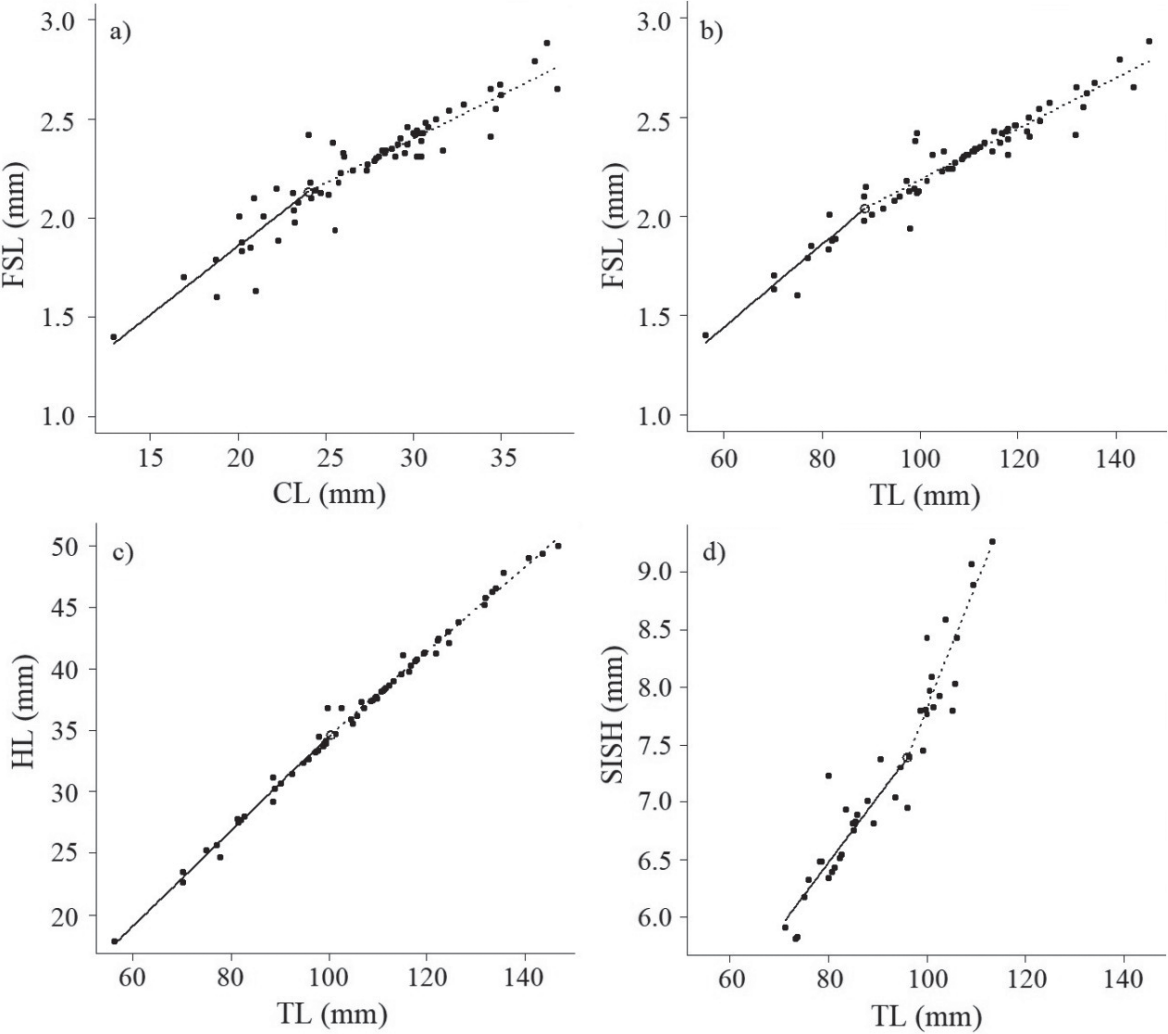
Break points estimated for morphometric relationships in females and males of *Solenocera acuminata*: Female: **a** first abdominal segment length (FSL) vs. cephalothorax length (CL) **b**FSL vs. total length (TL) **c** head length (HL) vs. TL; male: **d** sixth abdominal segment height (SISH) vs. TL.

A total of 68 females and 42 males were analysed separately in the break point analyses. The values shown in Table 3 correspond to those estimates that showed significant differences between the slopes (Davies’ Test, *P* < 0.05) and high coefficients of determination (r^2^ > 0.817), indicating a high correlation between sizes. For females, the slopes of the linear regression of the first segment were always greater than those of the second segment, while for the males, the slopes of the linear regression of the first segment were less than those of the second segment. The segmented regression with CL showed statistical significance only in females, with a break point of 23.80 ± 1.83 mm for FSL vs. CL. On the other hand, the segmented regressions, performed with TL as the main measure, were significant only for the FSL vs. TL and HL vs. TL relationships in females, showing break point values of 88.87 ± 4.92 mm and 99.85 ± 5.17 mm, respectively. For the males, a break point of 96.07 ± 33.3 mm was evident in the SISH vs. TL relationship (Table 3, Fig. 7a–d).

**Table 3.** The break point estimated by segmented regression for morphometric relationships of *Solenocera acuminata*: first abdominal segment length (FSL) vs. cephalothorax length (CL), FSL vs. total length (TL) and head length (HL) vs. TL for females; sixth abdominal segment height (SISH) vs. TL for males. The intercept and slope are presented for each segment.

Sex	n	Relationship	Break point (mm)	±SE	Segment	Intercept	Slopes	r^2^	Davies’ test *P*-value
F	68	FSL vs. CL	23.80	1.83	First	0.470	0.069	0.817	0.039*
					Second	1.078	0.044		
		FSL vs. TL	88.87	4.92	First	0.187	0.021	0.88	0.002*
					Second	0.911	0.013		
		HL vs. TL	99.85	5.17	First	-4.154	0.387	0.986	0.003*
					Second	0.295	0.343		
M	42	SISH vs. TL	96.07	3.33	First	1.914	0.057	0.906	0.018*
					Second	-2.832	0.106		

* Significant (*P* < 0.05).

**Figure 8.**
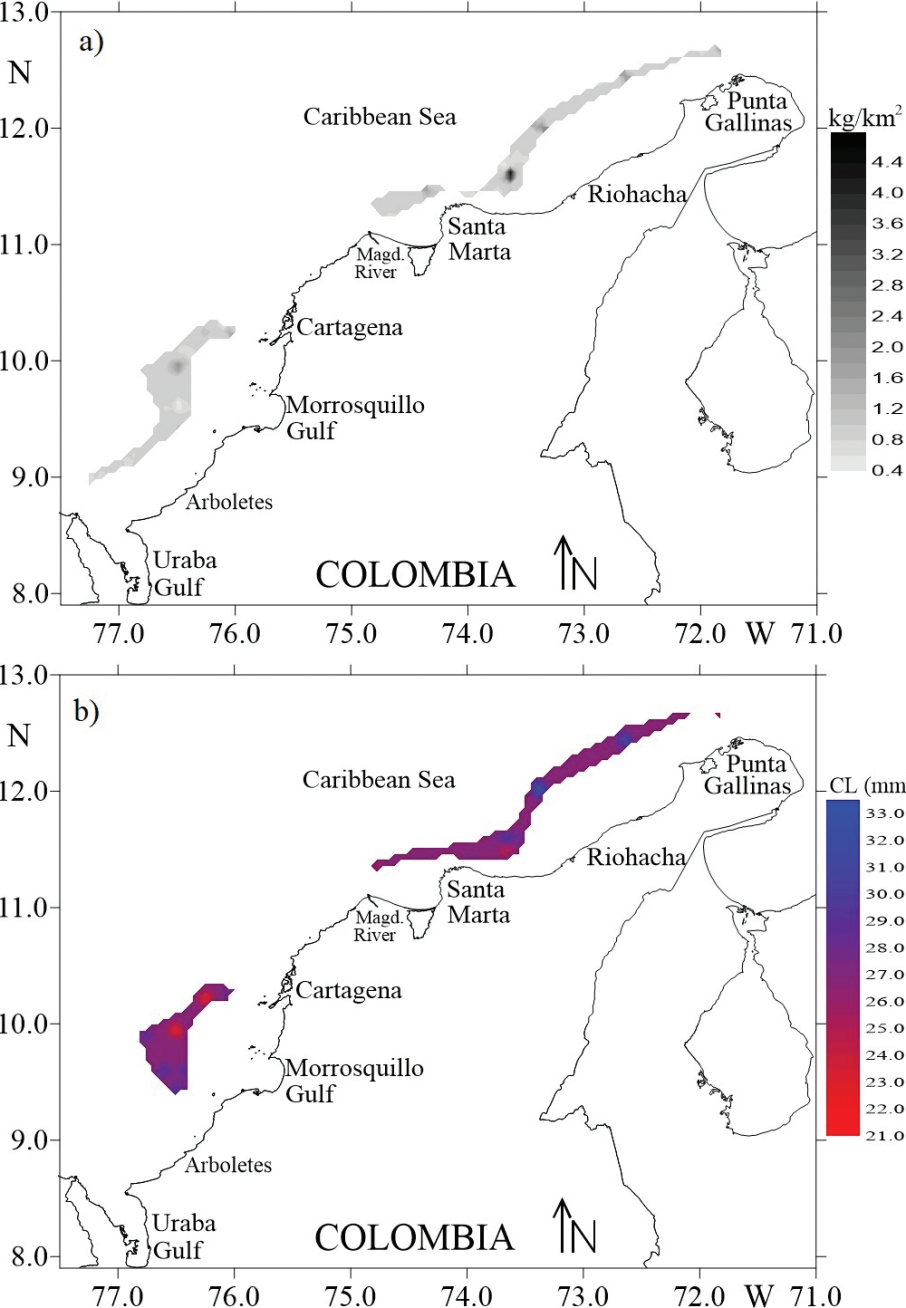
Spatial distribution of the **a** biomass (kg/km^2^) and **b** cephalothorax length (CL) (mm) of females *Solenocera acuminata* in the Colombian Caribbean.

The spatial structure of the biomass (kg/km^2^) of *S. acuminata* was modelled using an omnidirectional variogram, which is represented by a spherical model. The variogram showed a 59.33% nugget as a percentage of the sill (nugget = 0.39; sill = 0.27; range = 8.81 km). The spatial structure of the CL for females was also modelled by a spherical model. The variogram showed 0.00% of the nugget as a percentage of the sill (nugget = 0.00, sill = 10.72, range = 13.64 km). Relatively high biomass values were found in the northern zone of the Colombian Caribbean, between Santa Marta and Riohacha, where the mean biomass was 0.94 kg/km^2^ (coefficient of variation, CV = 39.97). In the southern zone, higher biomass was found between Cartagena and the Morrosquillo Gulf and the mean biomass in this zone was 0.89 kg/km^2^ (CV = 17.55) (Fig. 8). The spatial distribution of CL in females showed that the largest shrimp were found off the coast in the north and northwest of Riohacha and to the west of Punta Gallinas. The smaller individuals (~ 21–22 cm CL) in this study were found closer to the coast (~ 10 nautical miles, at 150 m of depth) to the northeast of Santa Marta. However, in the southern area, two aggregations of small individuals (~ 21–22 cm CL) were found off the coast between Cartagena and the Gulf of Morrosquillo (Fig. 8).

**Figure 9.**
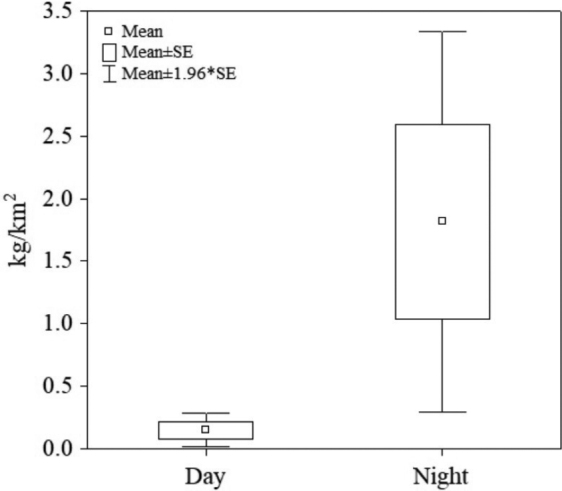
Box plot of the biomass (kg/km^2^) of *Solenocera acuminata* in the Colombian Caribbean differentiated by time of day.

The biomass of *S. acuminata* showed significant differences (*P* = 0.002) with the diel pattern, with higher values at night (mean 1.82 ± 3.81 kg/km^2^) than during daytime (mean 0.15 ± 0.37 kg/km^2^) (Fig. 9).

**Figure 10.**
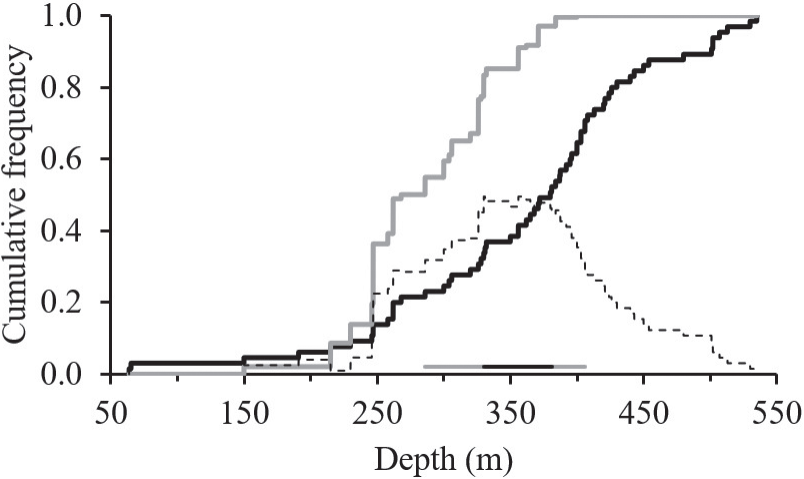
Cumulative density functions (CFDs) of the depth (f(t)) and the weighted biomass (kg/km^2^) of *Solenocera acuminata*. f(t) is shown by the thick black line, g(t) is shown by the thin grey line and the dotted line (d) is the absolute difference between g(t) and f(t). The depth preferences are shown as the grey and black straight lines.

The relationship between the biomass of *S. acuminata* and depth (m) showed significant associations (*P* < 0.01). This species was distributed between 150 and 400 m and the highest biomass was associated with depths ranging from 330.00 to 380.90 m (Fig. 10).

## Discussion

Information about the reproductive biology of a species is one of the most important aspects in the assessment of strategies for managing exploited populations (Li et al. 2012). The study of the reproductive biology in penaeid shrimp can facilitate our understanding of the adaptive strategies and reproductive potential of a species related to its environment (Gillett 2008). Although *S. acuminata* is of commercial interest, there are few studies about the biology of some species of the genus *Solenocera* and their roles in the ecosystems in which they are found (Demestre and Abelló 1993; Ohtomi and Irieda 1997; Ohtomi et al. 1998; Dineshbabu and Manissery 2008; Villalobos-Rojas and Wehrtmann 2011). The size at sexual maturity for females of *S. acuminata* (95.2 mm TL; 23.82 mm CL) is the first report for the species. The maximum TL in this study (females: 146.70 mm; males: 113.22 mm) are within the range of sizes recorded for females of a species of the same genus *Solenocera agassizii* in the Colombian Pacific (Rodríguez et al. 2012) and both sexes of this species in the Pacific Ocean in Costa Rica (Villalobos-Rojas and Wehrtmann 2011, 2018). Furthermore, the maximum CL values in this study (females: 38.17 mm; males: 27.97 mm) are similar to those of the same species in French Guiana (Guéguen 1997, 1998b) and slightly lower than those reported in the Colombian Caribbean Sea (Campos et al. 2005). However, they are within the range reported for the Western Atlantic (Pérez-Farfante and Bullis 1973) and French Guiana (Guéguen 1997, 1998b). For males, the maximum CL (27.97 mm) was within the ranges reported by previous authors (Pérez-Farfante and Bullis 1973; Guéguen 1997; Campos et al. 2005).

The mean size differences found between females and males are common amongst solenocerid shrimp (Dineshbabu and Manissery 2008; Li et al. 2012; Villalobos-Rojas and Wehrtmann 2018). The higher number of females than males in the larger size classes observed in *S. acuminata* (Fig. 4) has also been observed in other solenocerid species (Ohtomi and Irieda 1997; Dineshbabu and Manissery 2008; Li et al. 2012). The main factors that affect these variations in the sizes of females and males are differential longevity, mortality, migration behaviour and growth rates (Villalobos-Rojas and Wehrtmann 2018). Differences in the sizes of females and males may be due to differential mortality, nutrition restriction, greater activity of one sex, migration of one of the sexes at a given period and the use of different habitats by sex (Charnov and Hannah 2002; Chiba et al. 2006; Lizárraga-Cubedo et al. 2008; Baeza and Piantoni 2010; Grabowsky et al. 2014).

The highest biomass of *S. acuminata* was found in the northern zone of the Colombian Caribbean. The northeast trade winds drive the surface currents to the west and southwest, almost parallel to the coast, leading to Ekman transport away from the coast, which is responsible for upwelling in the northern zone of the study area and increased productivity along the Guajira coast (Andrade et al. 2003; Paramo et al. 2003, 2009; Correa-Ramírez et al. 2020). In fact, in the Guajira Region, high biomasses have been found for other deep-sea crustaceans, such as *Aristaeomorpha foliacea*, *Pleoticus robustus* (see Paramo and Saint-Paul 2012a), *Penaeopsis serrata* (see Paramo and Saint-Paul 2012b), *Metanephrops binghami* (see Paramo and Saint-Paul 2012c), *Glyphocrangon longleyi*, *Glyphocrangon neglecta* (see Pacheco et al. 2018) and *Agononida longipes* (see Espitia et al. 2019).

Morphometric relationships are an important factor for biological studies of fishery resources and stock assessment. In addition, for the management, it is very important to know the size structure, body growth and size at sexual maturity of this species (Hilborn and Walters 1992), all of which influence the structure and function of marine ecosystems (Haedrich and Barnes 1997; Shin et al. 2005). However, sometimes for practical reasons or due to body damage, only data from some body parts can be recorded (Zetina et al. 1996). Therefore, morphometric relationships have been established to calculate sizes and weight. The morphometric relationships analysed in this study can be useful for population studies of the same species in different geographic locations. The size structure, growth type and morphometric relationships are important parameters of life history and of great utility for the management of a new deep-sea fishery in the Colombian Caribbean. The most frequent dimensions used amongst the wide variety of morphometric measurements in penaeid shrimp are carapace length, total length, body width and wet weight (Özcan and Katağan 2011). Currently, studies on morphometric relationships in deep-water shrimp are scarce. However, these analyses indicate whether there are morphometric variations between several body measurements for the same species in a period of time (Kapiris 2005). These variations may be due to reproductive factors, since filled gonads can influence morphometry.

Morphometric analyses performed by Rudolph (1997, 1999, 2002) on other decapods indicated that, in *Samastacus spinifrons* and *Parastacus pugnax*, females have longer and wider abdomens than males. However, in this study, the first abdominal segment was shorter (Fig. 5i), but wider (Fig. 5j) in females than in males. In some male decapods, gonopods and abdominal segments do not increase in size faster than the carapace or the total length (Daniels 2001). However, females have a marked increase in size and changes in the shape of abdominal segments, especially the first abdominal segment, as well as pleopods and other parts of the body, which increases the area available for the fixation of eggs on the pleopods, acting as an incubation chamber for developing eggs (Daniels 2001). Therefore, it is necessary to study whether these growth patterns are related to different types of habitats, ecology, migration and reproduction. All the morphological variations, observed between the sexes, could be associated with differences in the growth pattern of females (e.g. larger maximum size and higher growth rate) compared to those of males (Kapiris 2005). Morphometric variations can be caused by evolutionary and environmental factors and genetic analysis should be used to confirm that these variations are associated with changes in reproductive morphology, rather than with environmental differentiation (Tzeng et al. 2001).

Penaeid shrimp usually show an allometric coefficient (*b*) close to 3. Female *S. acuminata* in the Colombian Caribbean followed an allometric growth pattern, which is consistent with previous studies regarding other decapods (Guéguen 1997; Josileen 2011; Özcan and Katağan 2011; Li et al. 2012). Males of *S. acuminata* followed an isometric growth pattern, as reported by Josileen (2011) and Segura and Delgado (2012) for other decapods. The length-weight relationship slope values of females in this study were similar to those reported in Kagoshima Bay, southern Japan, for *Solenocera melantho* (see Ohtomi and Irieda 1997) and higher than those reported for *Solenocera membranacea* in the coastal Aegean Sea of Turkey (Özcan and Katağan 2011) and *S. melantho* in the East China Sea (Li et al. 2012). Boschi (2016) reported allometric growth of other juvenile decapods in Argentina, such as *Pleoticus muelleri* and *Artemesia longinaris*, due to fluctuations in the relationship between different body parts. Several studies have shown similar results on sexual dimorphism in decapod crustaceans (Romero-Sedano et al. 2004; Faye et al. 2015; Ramírez et al. 2020), with females being larger than males and this sexual dimorphism is thought to be related to differences in the functions of male gonopods and female pleopods (Daniels 2001). In fact, after the complete development of gonopods, an isometric growth pattern or even a negative allometric pattern is reported (Fadlaoui et al. 2019). The growth pattern of some specific body parts, such as abdominal segment, gonopods and pleopods, shows variations in the degree of allometry during the course of the development, which may coincide with gonad maturation, providing an important estimate for the size at which these animals are ready for spawning (Marochi et al. 2016; Fadlaoui et al. 2019). However, most studies on the functional significance of isometric and allometric growth have been superficial and more care needs to be taken in explaining these biological phenomena (Daniels 2001). On the other hand, allometric analysis can also provide valuable information about evolutionary modifications in the growth of species (Tzeng and Yeh 2002).

Knowledge of the reproductive season and the average size at sexual maturity of a species with potential applications in fisheries is fundamental to designing and establishing monitoring and control strategies for its conservation. The spatial size structure of *S. acuminata* in the Colombian Caribbean was determined for the first time, which is interesting because it indicates possible breeding areas. However, for the Colombian Caribbean, there is currently no reproductive information available for the orange shrimp (*S. acuminata*). The analysis performed by Guéguen (1998a) on the continental slope of French Guiana revealed two seasons of sexual maturation of the gonads in females of *S. acuminata* (between May and June and between November and December). A very similar pattern arises in the species *S. agassizii*, which showed two spawning peaks per year from 2005 to 2011, one from May to July and another from December to January (Villalobos-Rojas and Wehrtmann 2018). However, these authors mentioned that this peak of high reproductive intensity fluctuates and is mainly associated with changes in water temperature and salinity, as well as food availability. The size at sexual maturity for females of *S. acuminata* (95.2 mm TL and 23.82 mm CL) is the first report for the species; these values serve as a reference point for this species in the Colombian Caribbean. In addition, an important factor should be considered in relation to the assignment of ovarian maturity stages performed in this work, wherein Stage II (early maturing) is classified as an immature female. Nevertheless, due to the sampling period, it is possible that females classified as being in Stage II had previously extruded a clutch of eggs and that their ovaries were in regeneration, which may cause confusion during classification. Incorrect classifications in our study may have affected the estimation of the maturity ogive. Moreover, it is recommended that, to validate these results, the future studies should use a histology analysis to obtain a more accurate ogive estimation (Flores et al. 2020). Therefore, it is recommended to carry out a monthly study to determine the reproductive characteristics of *S. acuminata*, such as its reproductive season and maximum reproductive potential; in addition, histological studies should be used to verify the stages of gonadal maturity and the activity of moults during the year to develop management measures, such as a temporal and/or spatial closure.

The discontinuities in the growth rates of some parts of the body in crustaceans may indicate variations in the morphological size of individuals at the onset of sexual maturity (Hall et al. 2006; Claverie and Smith 2009; Josileen 2011; Queirós et al. 2013; Severino-Rodrigues et al. 2016). The estimated maturity ogive in this study was associated with the results obtained in the break point analysis. The analysis of the first abdominal segment length (FSL) versus CL in the females in this study had relatively similar results as the analysis of the size at sexual maturity: FSL vs. CL = 23.80 mm CL (Table 3) and CL_50%_ = 23.82 mm (Fig. 6). These findings indicate that the variation in the length of the first abdominal segment, according to CL and TL, can be related to the morphological size at the onset of sexual maturity. Therefore, in this species, the growth of the first abdominal segment may be delayed until individuals reach CLs of 23.8 mm and TLs of 88.87 mm (Fig. 7a, b). This is the approximate time at which the onset of sexual maturity occurs (following a moult in puberty) and an abdominal morphology consistent with reproduction (i.e. a wider abdomen) and with the objective of incubating eggs is observed as occurring in other crustaceans (Rudolph 2002; Cusba and Paramo 2017). However, this change may be also associated with the post-maturing phase, when adult females with optimal abdominal sizes require energy for egg production, reducing the rate of abdominal growth (Claverie and Smith 2009). Therefore, evident changes in the size of the body area in the abdomen may be a good indicator of the beginning of morphological sexual maturity in *S. acuminata* females. However, the morphological size of the maturity (23.8 mm CL and 88.87 mm TL) is uncertain due to the inconsistencies that exist in the estimation of the maturity ogive presented in this study. For HL and SISH, several changes occur in decapods, particularly in the increase in the SISH (Boschi 2016). This increase could also be related to individuals that are close to mating, during which a thoracic-abdominal junction occurs from the male to the female, with the male facing the ventral regions of the female (Boschi 2016).

Guéguen (1997, 1998a) and Charbonnier et al. (2010) reported that *S. acuminata* is probably nocturnal-feeding, since it is captured only at night and burrowing during the day. This behaviour may be a crucial factor in the catch yields recorded during the present cruise, where daytime hauls showed significantly lower catches than night-time hauls. These animals rest when light increases and feed in darkness (Aguzzi and Company 2010). These patterns of diel periodicity agree with our results for *S. acuminata*, which also indicated a nocturnal feeding behaviour, as the largest catches were recorded during nocturnal trawls and burrowing behaviour during daylight. This behaviour should be considered in the sampling design of future surveys aimed at evaluating the population of *S. acuminata* in the Colombian Caribbean.

Size structure, size at sexual maturity, growth type and morphometric relationships presented in this work are initial reference parameters for fisheries managers. This important information could be useful and strengthened in future research in order to establish and implement management and conservation strategies for *S. acuminata*. Before starting a new fishery, more research is needed to understand the life cycle parameters of this deep-sea resource, such as its growth, reproduction, recruitment, mortality, spawning areas and times, nursery areas and associated biodiversity.
